# Evidence of electronic polarization of the As ion in the superconducting phase of F-doped LaFeAsO

**DOI:** 10.1107/S2052252514005636

**Published:** 2014-04-14

**Authors:** Jungeun Kim, Akihiko Fujiwara, Tomohiro Sawada, Younghun Kim, Kunihisa Sugimoto, Kenichi Kato, Hiroshi Tanaka, Motoyuki Ishikado, Shin-ichi Shamoto, Masaki Takata

**Affiliations:** a Japan Synchrotron Radiation Research Institute, Kouto, Sayo-cho, Hyogo 679-5198, Japan; b RIKEN SPring-8 Center, Kouto, Sayo-cho, Hyogo 679-5148, Japan; c Department of Advanced Materials Science, The University of Tokyo, Kashiwa, Chiba 277-8561, Japan; d Department of Materials Science, Shimane University, 1060 Nishi-kawatsu-cho, Matsue, Shimane 690-8504, Japan; e Comprehensive Research Organization for Science and Society (CROSS), Ibaraki 319-1106, Japan; f Japan Atomic Energy Agency, Ibaraki 319-1195, Japan

**Keywords:** superconductivity, electronic polarization, charge-carrier redistribution

## Abstract

Understanding the nature of superconductivity in iron-based compounds is essential in the development of new strategies to increase *T*_c_. Using a charge density analysis based on synchrotron radiation X-ray powder diffraction data, we found that the charge carriers only accumulated in the iron layer of the superconducting phase of LaFeAsO_1 − *x*_F*_x_* at low temperatures. Analysis of the electrostatic potential distribution revealed the concerted enhancement of the electronic polarization of the As ions and the carrier redistribution. This suggests that the enhanced electronic polarization of the As ion plays an important role in inducing high *T*_c_ superconductivity, and that the polaron concept, which has been previously regarded as an untenable mechanism, should be reconsidered for the description of the iron–arsenide superconducting phase.

## Abstract 2

Charge-carrier redistribution invoked by enhanced electronic polarization of the As ion was observed in the superconducting phase of iron arsenide.

## 1. Introduction   

The recent discovery of new superconducting phases in iron-based materials (Kamihara *et al.*, 2008 ▶; Nomura *et al.*, 2008 ▶; Iimura *et al.*, 2012 ▶) has compelled the reconsideration of the roles that charge, spin and lattice degrees of freedom play in superconductivity. For most superconducting phases observed at low carrier densities, it was previously thought that spin fluctuation plays an important role in the onset of superconductivity, in which nesting between hole and electron pockets is the origin of Cooper-pair formation; this is enhanced when the FeAs tetrahedral structure forms an ideal tetrahedron and the pnictide height is about 1.39 Å (Lee *et al.*, 2008 ▶; Kuroki *et al.*, 2009 ▶). Recently, a new superconducting phase in the high-carrier-density region was discovered, and an orbital fluctuation mechanism involving the degeneration of Fe 3*d* bands was proposed as the cause of the pairing (Iimura *et al.*, 2012 ▶). Thus the debate on the mechanism of superconductivity in iron-based materials has been reignited because of these various proposed pairing mechanisms. Here, by means of a precise charge-density study *via* synchrotron radiation X-ray powder diffraction, we report a new feature, electronic polarization, associated with carrier accumulation that is only observed in the superconducting phase of LaFeAsO_1 − *x*_F*_x_*. We propose that electronic polarization is another key factor in the onset and/or enhancement of superconductivity in iron-based materials, in addition to spin and orbital fluctuations.

## 2. Experiment   

Powder samples of LaFeAsO_1 − *x*_F*_x_* (*x* = 0–0.200) were synthesized by solid-state reaction using Fe_2_O_3_, Fe, LaAs and FeF_2_ powders as starting materials in evacuated silica tubes. The fluorine contents, *x*, of the samples were determined by secondary ion-microprobe mass spectrometry. The superconducting transition temperatures (*T*_c_) were characterized as the onset temperatures (*T*_c_^onset^) of the Meissner signals by superconducting quantum interference device (SQUID) measurement. The X-ray powder diffraction measurements on the powder samples of LaFeAsO_1 − *x*_F*_x_* (*x* = 0–0.200) were carried out at Powder diffraction beamline BL02B2 and RIKEN Materials Science beamline BL44B2 of SPring-8 to obtain the patterns of high statistics (∼ 3 × 10^5^ counts) with high angular resolution (*d* < 0.5 Å). The powder samples were sealed in a 0.3 mm glass capillary. The temperature was controlled by an *N*_2_ gas flowing system at 90–300 K and a helijet system (Agilent Technologies) below 90 K. The wavelength of incident X-rays was 0.4999 (1) Å. The diffraction patterns were collected for 3–5 min on an imaging plate installed on a large Debye–Scherrer camera. Using the structural model obtained by Rietveld refinement, we have determined the precise structure parameters such as lattice constant, bond length, bond angle and interlayer distance upon F-doping. For analysis of electron density structure by means of Maximum Entropy Method (MEM), a total of 352 observed structure factors were extracted after Rietveld refinement; the MEM analysis of each composition was carried out with the program *ENIGMA* using 128 × 128 × 128 pixels (Sakata *et al.*, 1990 ▶; Takata & Sakata, 1996 ▶). The charge density obtained was converted to the electrostatic potential *U*(**r**) (Tanaka *et al.*, 2006 ▶). *U*(**r**) is divided into the two components, that is, the nucleus charge component [*U*_n_(**r**)] and the electron charge component [*U*_e_(**r**)]. *U*_n_(**r**) is estimated by the ordinary Ewald’s method, while *U*_e_(**r**) is directly calculated from the MEM charge density. The electrostatic potential obtained was visualized using the OpenDX program provided by IBM Visualization Data Explorer.

## 3. Results and discussion   

The crystal structure of LaFeAsO is composed of an alternately stacked structure of FeAs_4_ conducting layers and La_4_O blocking layers. The undoped parent compound has a structural phase transition from a tetragonal phase to an ortho­rhombic phase near 150 K upon cooling, and it undergoes antiferromagnetic spin-density-wave (SDW) ordering (Kamihara *et al.*, 2008 ▶; Nomura *et al.*, 2008 ▶). With F-doping on the oxygen sites, this structural phase transition and antiferromagnetic formation are suppressed and superconductivity is exhibited (Fig. S1). To determine the structural dissimilarity between the superconductor and the non-superconductor, we have analyzed the powder diffraction data for LaFeAsO_1 − *x*_F*_x_* using the MEM/Rietveld method (Sakata *et al.*, 1990 ▶; Takata & Sakata, 1996 ▶). The results show that the fundamental structural parameters simply follow Vegard’s rule (Denton & Ashcroft, 1991 ▶). The bond angle and the pnictide height of the FeAs tetrahedron, which have been considered as key parameters in iron-based superconducting materials, linearly decreased and increased, respectively, with F-doping. Other structural parameters such as the bond length and the bond angle of the LaO layer as well as the distance between FeAs and the LaO layer also displayed linear relationships with F-doping (Figs. S3 and S4). The fundamental structural parameters were in very good agreement with previously reported values (Kamihara *et al.*, 2008 ▶; Nomura *et al.*, 2008 ▶) and exhibited clear deformation of the FeAs tetrahedron as a result of F-doping. There existed no clear correlation, however, between the structural parameters at the atomic position level and *T*_c_ of this system.

**Figure 1:**
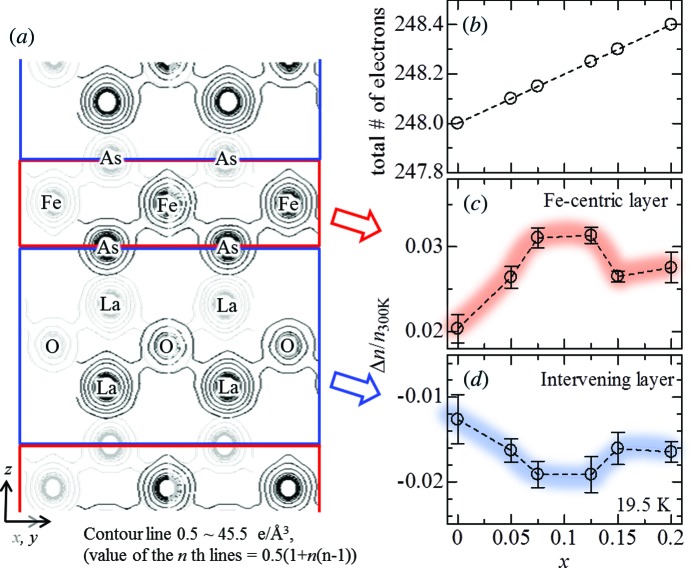
F-concentration (*x*) dependence of the total number of electrons (*b*), and changes in the electron number within the Fe-centric layer (*c*) and intervening layer (*d*) at 19.5 K. (*a*) shows the contour map of MEM charge density at (100) (black contour lines) and (010) plane (grey contour lines) of LaFeAsO_0.95_F_0.05_. To evaluate the degree of electron accumulation within the Fe layer, the computing region was divided into two layers on the basis of the As site. Δ*n* (≡ *n* − *n*_300 K_, where *n* is the electron number within a layer) on each layer was normalized by the electron number (*n*_300 K_) at room temperature.

**Figure 2:**
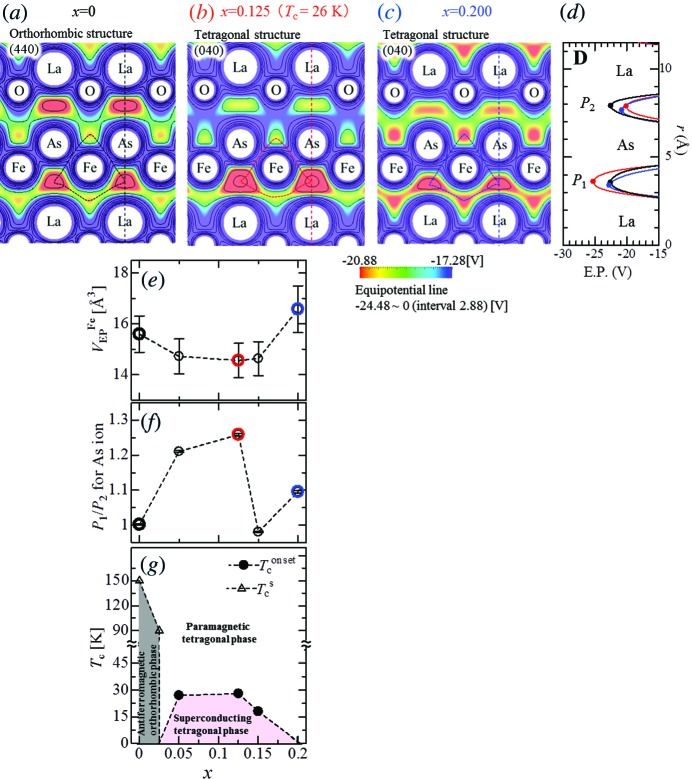
Electrostatic potential map with equipotential lines (*a*)–(*c*), variation of the electrostatic potential volume (*V*_EP_^Fe^) of the Fe atom (*e*) and asymmetric ratio (*P*_1_/*P*_2_) of electrostatic potential distribution around the As ion (*f*) as a function of F-content (*x*) at 19.5 K. *T*_c_ are plotted in (*g*). The dashed curve around Fe in (*a*)–(*c*) is the trace of the local minimum points of electrostatic potential around the Fe atom. The minimum points, *P*_1_ (*P*_2_), of electrostatic potential distribution around the As ion are shown in (*d*) which is the one-dimensional plot of the dashed line crossing the center position of the La and the As ion. *T*_c_^onset^ and *T*_c_^s^ in (*g*) are defined by the onset temperatures of the Meissner signals and the temperatures of the structural phase transition from a tetragonal phase to an orthorhombic phase.

In contrast to conventional analyses of the structural parameters, the MEM charge density, which was obtained directly using the integrated intensities of the X-ray diffraction pattern, showed significant changes in the superconducting phase. Fig. 1 ▶ shows the total number and changes in the number of electrons at 19.5 K relative to that at 300 K for the Fe-centric layer and the intervening layer. Whereas the total number is assumed to increase monotonically as the F-concentration (*x*) increases (Fig. 1 ▶ *b*) and no temperature dependence is expected, the distribution of electrons in each layer in the unit cell depends strongly on *x* and *T*, being enhanced at the values of *x* at which superconductivity occurs; at 19.5 K, the change in the number of electrons in the Fe-centric layer increased with *x* and showed a maximum around *x* = 0.100 (Fig. 1 ▶ *c*). Trends in the intervening layer were opposite to those in the Fe-centric layer (Fig. 1 ▶ *d*). At low temperatures, this fascinating feature is supposedly caused by the accumulation of electrons in the Fe layer and was confirmed by the electrostatic potential analysis of the MEM results (Tanaka *et al.*, 2006 ▶; Kim *et al.*, 2011 ▶; Fujiwara *et al.*, 2012 ▶). Figs. 2 ▶(*a*)–(*c*) show two-dimensional maps with equipotential lines. Unlike the MEM charge density, which may be affected by hybridization, the electrostatic potential allows the volume (*V*_EP_) occupied by each atom to be estimated (Fujiwara *et al.*, 2012 ▶). The three-dimensional volume is formed by traces of the local minimum point of the electrostatic potential. According to the electrostatic potential formula, the boundary of *V*_EP_ is placed in a quasi-neutral point; an increase in the number of electrons causes a shrinkage of *V*_EP_ to maintain neutral character in the boundary of electrostatic potential. We focused on variations in the *V*_EP_ of the Fe atom (*V*_EP_^Fe^), as the behavior of the Fe atom is thought to be closely linked to superconductivity in this system. At 19.5 K, *V*_EP_^Fe^ shrank by over 7% at *x* = 0.050, but it then increased at *x* = 0.150 (Fig. 2 ▶ *e*). This large shrinkage cannot be attributed to a decrease in the lattice volume alone, which resulted in at most a 1.3% decrease at *x* = 0.200 (Table S1). The variation in *V*_EP_^Fe^, therefore, reflects that electrons are effectively concentrated in the Fe layer in the 0.050 ≤ *x* ≤ 0.150 region at this low temperature.

Along with this electron charge redistribution in the Fe layer, some notable features have been observed in the variations of the local electrostatic potential. At *x* = 0.125, the equipotential lines showed distinct differences in the upper and lower parts of the As ion, compared with isotropic variations at *x* = 0 and 0.200; the upper equipotential lines show a gradual slope with smooth curves, in comparison to the closely spaced lower equipotential lines. These effects lead to an extremely asymmetrical distribution of the electrostatic potential around the As ion, which arises from a separated electrostatic potential distribution on the As ion (Fig. S5). Such a separated electrostatic potential distribution on an atom was observed from electrostatic potential imaging of PbTiO_3_ by Tanaka *et al.* (2006 ▶), who reported that the specific separation of the electrostatic potential distribution is a signature of polarization. Enhancement of the electronic polarization induces a large change in the electrostatic potential distribution because of its influence on the electrostatic interactions between electrons distributed in that space and those on the As ion. In order to estimate the degree to which the electronic polarization of the As ion was enhanced, we evaluated the asymmetric ratio, *P*_1_/*P*_2_, where *P*_1_ (*P*_2_) is the minimum value of electrostatic potential between the As ion and lower (upper) La ion. In the superconducting phase (*x* = 0.125), *P*_1_ and *P*_2_ were respectively −25.24 and −20.06 V (*P*_1_/*P*_2_ = 1.258) (Fig. 2 ▶ *d*). A noticeable difference was not observed at *x* = 0 (*P*_1_/*P*_2_ = 1.002) and *x* = 0.200 (*P*_1_/*P*_2_ = 1.096), which were not the superconducting phase (Fig. 2 ▶ *f*). It is worth noting that the electronic polarization of the As ion is significantly enhanced in the superconducting phase, which was clearly shown by the asymmetric contour curves and the resultant asymmetrical spatial distribution of the electrostatic potential around the As ion.

**Figure 3:**
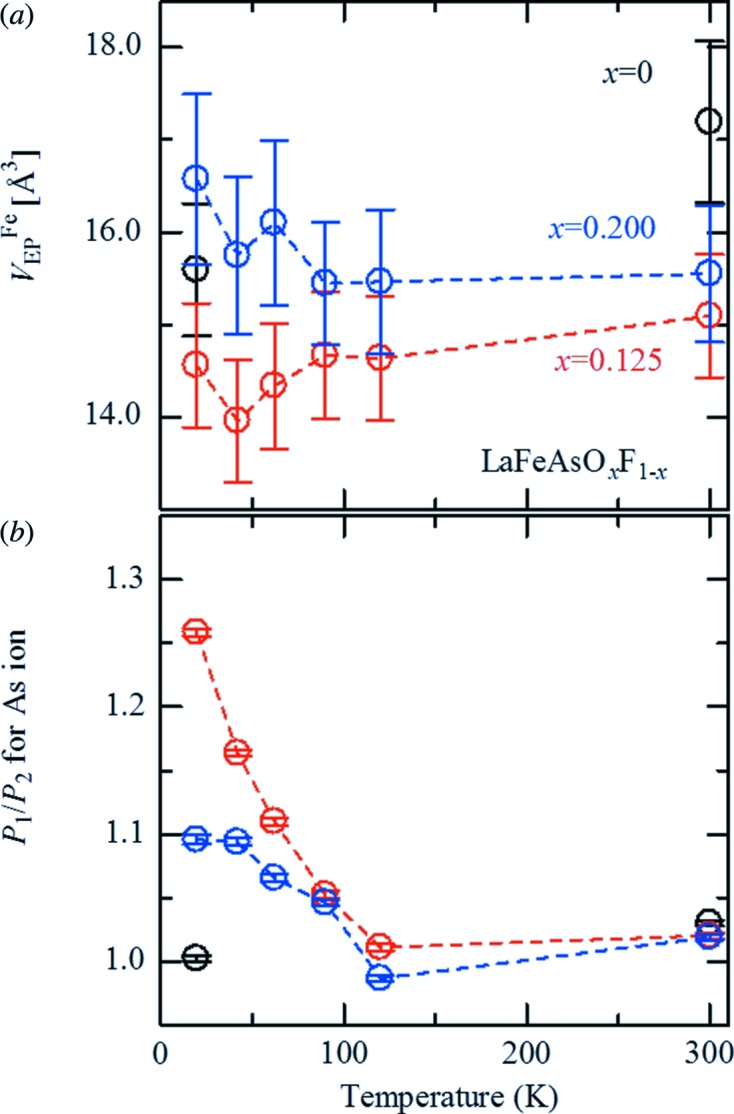
Temperature dependence of the electrostatic potential volume (*V*_EP_^Fe^) of the Fe atom (*a*) and the degree of asymmetric space distribution (*P*_1_/*P*_2_) around the As ion (*b*) at *x* = 0.125 (red circles) and *x* = 0.200 (blue circles). Black circles are the data for *x* = 0 at 19.5 and 300 K.

Both *V*_EP_^Fe^ and the asymmetric ratio of the electrostatic potential on the As ion vary not only with F-concentration (*x*) but also with temperature, as shown in Fig. 3 ▶. At room temperature for *x* = 0.125, *V*_EP_^Fe^ was reduced significantly by 13%, indicating that surplus electrons from F-doping are confined in the Fe layer at 300 K (Fig. 3 ▶ *a*). However, no further shrinkage of *V*_EP_^Fe^ was observed at *x* = 0.200, which suggests that the number of electrons that can accumulate in the Fe layer reaches its maximum for *x* = 0.125 at 300 K, although the total number of electrons continues to increase as *x* increases. After cooling to 19.5 K, however, *V*_EP_^Fe^ for *x* = 0.125 shrank by a further 7%, indicating further electron accumulation. Although *V*_EP_^Fe^ for *x* = 0 also shrank with decreasing temperature, at 19.5 K it had not reached the value for *x* = 0.125 at 300 K; an insufficient number of electrons accumulated for superconductivity. *V*_EP_^Fe^ at *x* = 0.200, on the other hand, increased by ∼ 7%, showing that electron numbers in the Fe layer decreased with cooling. To account for the large contraction of *V*_EP_^Fe^ at *x* = 0.125, an additional effect, which can outweigh the less-than-1% shrinkage of the lattice volume, is required at low temperature. As well as the marked contraction of *V*_EP_^Fe^, *P*_1_/*P*_2_ for the As ion increased considerably at *x* = 0.125, for which superconductivity was observed under 26 K (Fig. 3 ▶ *b*). As the temperature decreased, *P*_1_/*P*_2_ scarcely changed for *x* = 0. *P*_1_/*P*_2_ of both doped samples, on the other hand, began to increase near 90 K, and *P*_1_/*P*_2_ for *x* = 0.125 rose significantly to a value of 1.26 until the temperature had fallen to 19.5 K, whereas that for *x* = 0.200 increased no further than 1.1 at 42 K.

Based on *V*_EP_^Fe^ and *P*_1_/*P*_2_, we can understand the effect of doping on the emergence of superconductivity as follows. At 300 K surplus electrons from F-doping are confined to the Fe layer and the electronic polarization of the As ion is not changed. As temperature decreases from near 70 K, the electronic polarization of the As ion is only significantly enhanced in the superconducting phase, and the accumulation of electrons in the Fe layer is accelerated. Consequently, our results suggest that the electronic polarization of the As ion is one of the crucial factors in inducing superconductivity in LaFeAsO_1 − *x*_F*_x_*. We postulate that this electronic polarization is the average result, as observed by the steady-state X-ray diffraction, of polaronic behavior, which is a dynamic motion. Until now, electron-phonon coupling has been regarded as an untenable mechnism in high *T*_c_ iron-based superconductivity, because a *T*_c_ value beyond the McMillan limitation (McMillan, 1968 ▶) could not be explained by such a pairing mechanism (Kamihara *et al.*, 2008 ▶; Nomura *et al.*, 2008 ▶; Iimura *et al.*, 2012 ▶). In spite of this weakness, an additional effect related to polarization has been consistently proposed in iron superconductivity. Recently Sawatzky *et al.* has suggested a bipolar­onic concept (Sawatzky *et al.*, 2009 ▶; Berciu *et al.*, 2009 ▶), in which the fluctuation of electrons on the Fe ion leads to the electronic polarization of the As ion; the polarized electron clouds interact attractively, and thereby propagate effective charge carriers into the Fe layer. According to the theory, the As 4*p*-orbital is polarized toward the Fe atom by residual electrons on the Fe atom. As shown in Figs. S5 and S6, the electronic polarization of the As ion is, as an average of the dynamic fluctuation, in the direction of the *c*-axis. This is very likely indicative of a polaron or bipolaron; the in-plane component of the polaron was canceled out in the time-averaged XRD results, and the polarization induced by the bipolaron was observed along the *c*-direction. Although only three cases have been computed and the many-body problem still remains, the direction of electronic polarization being aligned toward the *c*-axis, which has been visualized by electrostatic potential imaging, is reasonable evidence for the existence of polaron/bipolaron, in addition to the enhanced electronic polarization only occurring in the superconducting phase. As well as the specific direction and emergence of the electronic polarization of the As ion in the superconducting phase, the onset temperature of electronic polarization also suggests the existence of polaron. Recently, Zhang *et al.* (2010 ▶) measured the Fe—As mean-square bond displacements (σ^2^_As—Fe_) in LaFeAsO_1 − *x*_F*_x_* (*x* = 0 and 0.07) using X-ray absorption fine-structure techniques. Generally, the σ^2^_As—Fe_ values decreased as the temperature decreased due to the suppressed thermal vibration of the atom. However, σ^2^_As—Fe_ for *x* = 0.07, for which superconductivity appears at 26 K, decreased with cooling at temperatures above 70 K, but it then increased abruptly near 70 K. This anomaly reached a maximum near 30 K, while σ^2^_As—Fe_ for *x* = 0 decreased continuously as the temperature decreased, and the same value remained almost constant at temperatures below 150 K. It was proposed that the abnormal enhancement of σ^2^_As—Fe_ was due to polaron formation in the superconducting phase of LaFeAsO_0.93_F_0.07_. The polarization of the As ion seen here, shown as *P*_1_/*P*_2_ in Fig. 3 ▶(*b*), is in good agreement with this result. That is, the asymmetric spatial distribution can be related to the distorted electron clouds in the FeAs layers, and implies that superconductivity in this system can be ascribed to polarons caused by electronic polarization of the As ion. The good correspondence between the carrier accumulation in the Fe layer, the electronic polarization of the As ion, and the appearance of superconductivity suggests that the electronic polarization of the As ion and carrier accumulation are cooperative factors in the emergence of iron–arsenide superconductivity.

## 4. Summary   

In summary, we have visualized the electronic polarization of the As ion within LaFeAsO_1 − *x*_F*_x_ via* MEM charge density and electrostatic potential methods using synchrotron radiation X-ray powder diffraction data. It was found that enhanced electronic polarization of the As ion is one of the most important factors, along with the accumulation of carriers in the Fe layer, for the onset of high *T*_c_ superconductivity. Moreover, we have demonstrated that the polaron mechanism mediated by electronic polarization of the As ion together with characteristics of the Fe ion should not be overlooked in the analysis of iron–arsenide superconductivity.

## Supplementary Material

Additional results. DOI: 10.1107/S2052252514005636/yc5002sup1.pdf
