# Supplementary material for: Evidence of electronic polarization of the As ion in the superconducting phase of F-doped LaFeAsO
Source: IUCrJ. 2014 Apr 14;1(Pt 3):155–9. doi: 10.1107/S2052252514005636 (PMC4086433; doi:10.1107/S2052252514005636)

IUCrJ

Volume 1 (2014)

Supporting information for article:

Evidence of electronic polarization of the As ion in the superconducting phase of F-doped LaFeAsO

Jungeun Kim, Akihiko Fujiwara, Tomohiro Sawada, Younghun Kim, Kunihisa Sugimoto, Kenichi Kato, Hiroshi Tanaka, Motoyuki Ishikado, Shin-ichi Shamoto and Masaki Takata

SUPPORTING INFORMATION for

Evidence of electronic polarization of the As ion in the superconducting phase of F-doped LaFeAsO

S1. Electrical resistivity of $LaFeAsO_{1-x}F_x$

The temperature dependence of the electrical resistivity is shown in **Fig. S1**. The parent compound, LaFeAsO, shows an anomaly of electrical resistivity near 150K, associated with the formation of antiferromagnetic spin-density-wave (SDW). The anomaly disappears in 5% F-doped LaFeAsO and superconducting transition occurs.

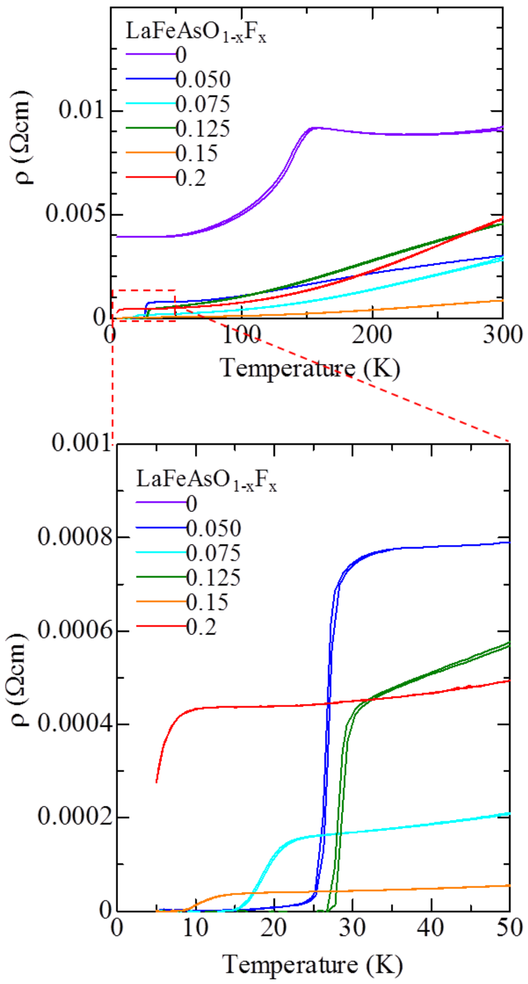

Figure S1 Temperature dependence of the electrical resistivity of $LaFeAsO_{1-x}F_x$.

S2. Structural parameters

We summarized the structural parameters in the superconducting phase (x = 0.125) and the non-superconducting phase (x = 0 and 0.200) of $LaFeAsO_{1-x}F_x$ at 19.5 K obtained by MEM/Rietveld analysis as shown in **Table S1 and S2**. **Fig. S2** shows the result of Rietveld analysis of x = 0.125 at 19.5 K for a reference of typical Rietveld result with raw data.

Table S1 Space group, lattice parameter (a, b, c), lattice volume (V), and reliability factors (R_I (R_{wp}), R_{MEM}) for Rietveld and MEM analysis of x = 0, 0.125, and 0.200 at 19.5 K.

Compound	x = 0	x = 0.125	x = 0.200
Formula	LaFeAsO	$LaFeAsO_{0.875}F_{0.125}$	$LaFeAsO_{0.8}F_{0.2}$
Space group	*Cmma*	*P*4/*nmm*	*P*4/*nmm*
a (Å)	5.70680(2)	4.01524(1)	4.01253(1)
b (Å)	5.67857(2)	4.01524(1)	4.01253(1)
c (Å)	8.71612(4)	8.68783(4)	8.66219(6)
V (Å^3)	282.459(3)	140.067(1)	139.465(2)
Z	4	2	2
R_I (R_{wp}) (%)	1.92 (4.55)	1.62 (4.38)	1.45 (4.69)
R_{MEM} (%)	2.22	1.79	1.76

$$R_I \equiv \frac{\sum_{hkl}\left|I_{hkl} - I_{hkl,calc}\right|}{\sum_{hkl} I_{hkl}}, R_{wp} \equiv \left[\frac{\sum_i w_i\left(y_i - y_{i,calc}\right)^2}{\sum_i w_i {y_i}^2}\right]^{\frac{1}{2}}, R_{MEM} \equiv \frac{\sum_K\left|F_{obs}(K) - F_{calc}(K)\right|}{\sum_K F_{obs}(K)}$$

where w_i is the weight, y_i ($y_{i,calc}$) is the observed (calculated) intensity at step i, $I_{hkl} = mF^2_{hkl}$ (m is the multiplicity, F_{hkl} is the structure factor).

Table S2 Atomic positions (*X*, *Y*, *Z*) and isotropic atomic displacement parameter (*B*) for *x* = 0, 0.125, and 0.200 at 19.5 K.

x = 0	site	*X*	*Y*	*Z*	*B*
La	4*g*	0	1/4	0.14186(4)	0.06(1)
Fe	4*b*	1/4	0	1/2	0.15(2)
As	4*g*	0	1/4	0.65144(8)	0.10(1)
O	4*a*	1/4	0	0	0.11(6)

x = 0.125	site	*X*	*Y*	*Z*	*B*
La	2*c*	1/4	1/4	0.14716(4)	0.14(1)
Fe	2*b*	3/4	1/4	1/2	0.10(1)
As	2*c*	1/4	1/4	0.65204(7)	0.06(1)
O	2*a*	3/4	1/4	0	0.20(6)

x = 0.200	site	*X*	*Y*	*Z*	*B*
La	2*c*	1/4	1/4	0.15002(5)	0.31(1)
Fe	2*b*	3/4	1/4	1/2	0.18(2)
As	2*c*	1/4	1/4	0.65264(10)	0.13(1)
O	2*a*	3/4	1/4	0	0.25(9)

Figure S2 The result of Rietveld analysis of x = 0.125 at 19.5 K. The red points are the synchrotron X-ray powder diffraction data. The blue line is the calculated pattern by the Rietveld refinement. The black lines show the Bragg peak positions and the green line is the difference between observed data and calculated pattern.

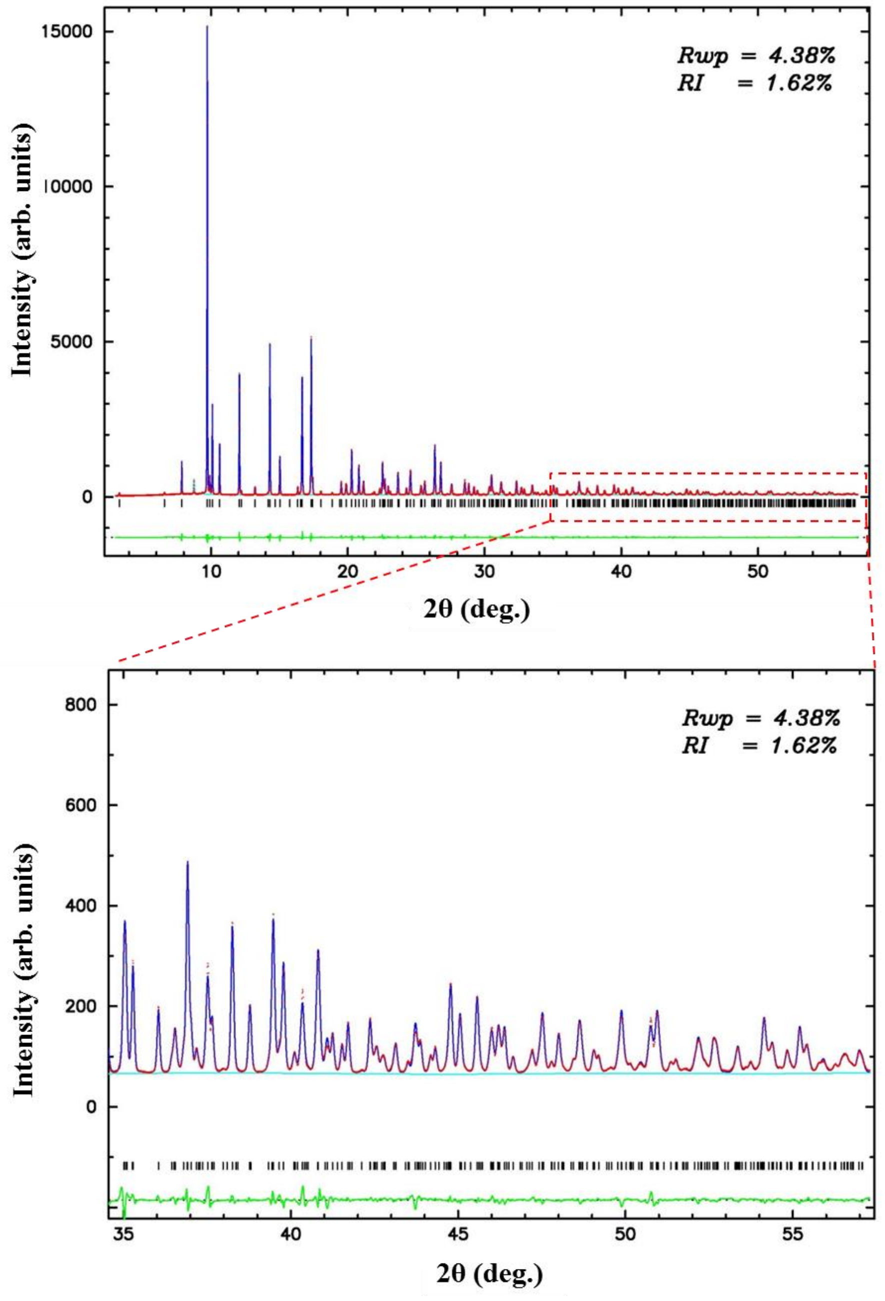

S3. The relation between structural parameters and T_c

The bond lengths of the nearest-neighboring (*nn*) La-As, Fe-As, and La-O(F) were plotted in **Fig. S3** as a function of temperature. The *nn*La-As bond length decreased with increasing F-content (x), and it decreased slightly with decreasing temperature. The bond length of the Fe-As shrunk slightly with cooling, and it didn't depend on the F-doping, while that of the La-O(F) showed a monotonic increase with F-doping. In order to investigate the relation between T_c and structural parameters (α(β) and h_{As}), which have been known as key parameters for superconductivity in the iron-based family, data were plotted as a function of F-concentration (x) at 19.5 K and 300 K as described in **Fig. S4**. In previous reports, the value of α/β (h_{As}) for a high symmetry tetrahedron was suggested as optimum value for high T_c superconductivity in the iron-arsenide system. The iron arsenic tetrahedral structure in this system, however, formed a higher symmetry at x = 0.200 where superconductivity disappeared. In $LaFeAsO_{1-x}F_x$, T_c had no correlation with the structural parameters at the atomic position level.

Figure S3 Temperature dependence of the bond length of the nearest-neighboring (*nn*) La-As (**A**), Fe-As (**B**), and La-O(F) (**C**).

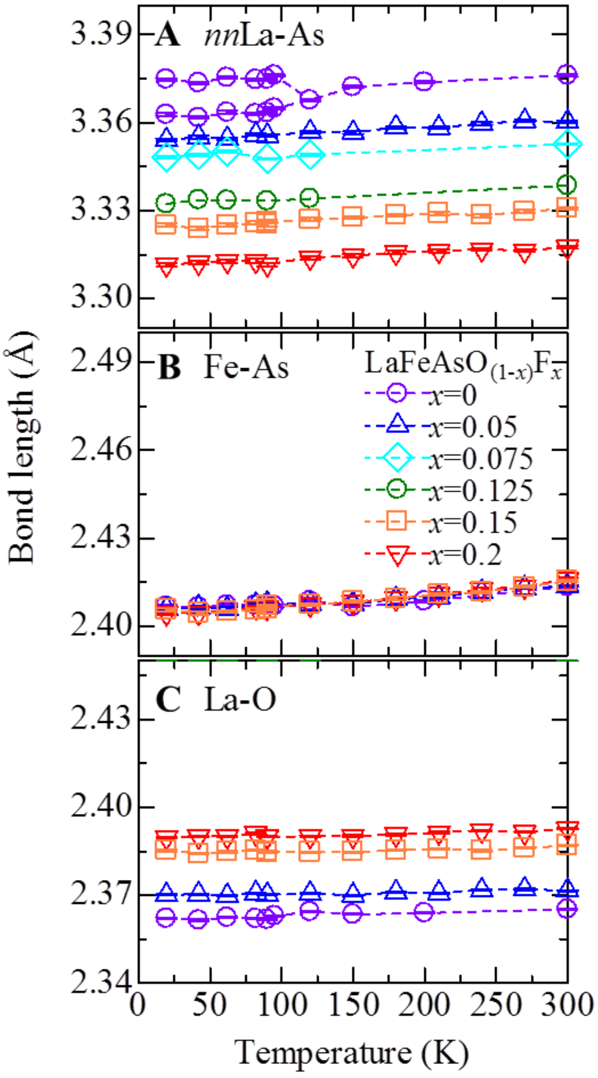

Figure S4 F-content (x) dependence of the bond angle and the pnictide height of the $FeAs_4$ tetrahedral structure at 19.5 K (blue circles) and 300 K (red circles). The left schematic picture is the $FeAs_4$ tetrahedral structure. It shows the bond angle of the As-Fe-As (α and β) and the pnictide height (h_{As}) on the Fe plane within the FeAs tetrahedral structure. Suggested optimum values for α/β and h_{As} were plotted as dashed lines.

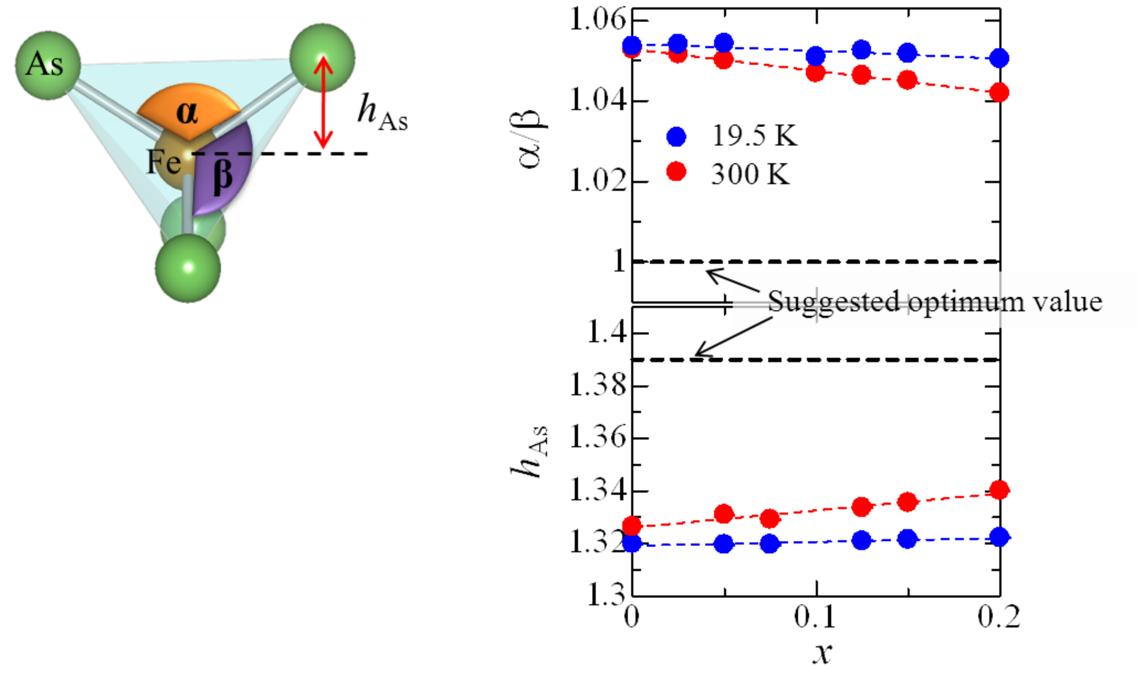

S4. Error estimation

The MEM is a well-established method that provides the most likely results based on statistical mechanics and information theory from conserved datasets; however, errors for the analyzed data cannot be extracted owing to the principle on which it is based. For reference, the reliability factor R_{MEM} is an indicator with which to evaluate the validity of the results; the values were less than 3 % for all data, showing the good reliability of the results. The values estimated from the MEM results, on the other hand, include errors due to the finite pixel size, and the error bars originating from this ambiguity have been shown for all data. In order to estimate the errors caused by determination of pixel assignment of local minimum position and atom basis, in all data of the MEM and the electrostatic potential of the manuscript and the supplementary information, we have applied methods of error bar computation as follows.

A) The error bars of **Figure 1C** and **1D** were estimated by a difference of the electron numbers within volume which is specified by increase/decrease of 1pixel at pixel position of the As basis.

B) In the **Figure 2E** and **3A**, the volume errors were assessed by surface area, which is accessed by tracing pixels with the minimum point of the electrostatic potential.

C) The error bars of **Figure 2F** and **3B** were estimated by the value difference of the electrostatic potential between at the pixel of the local minimum value and at its near-nearest pixel.

S5. A clear separation of the electrostatic potential distribution on the As ion in the superconducting phase.

The enhanced electronic polarization feature of the As ion is shown in a clear separation of the electrostatic potential in the tridimensional figure (**Fig. S5**) as well as an asymmetric distribution of the electrostatic potential in the two-dimensional map described in the manuscript; at x = 0.125 where the effective electrons were significantly accumulated in the Fe layer, electrostatic potential distribution on the As ion was clearly separated with the value of 28.8 V (red colored region) and that of 10.8 V (green colored region) along the c-axis as shown in **Fig. S5**. The similar separation of electrostatic potential on the As ion seemed to exist in the sample with x = 0, but the degree of separation was weaker than that for x = 0.125. The separation becomes more vague at x = 0.200.

Figure S5 Electrostatic potential distribution of the FeAs layer in $LaFeAsO_{1-x}F_x$ (x = 0, 0.125, 0.200) at 19.5 K. The tridimensional figures were colored by the value of the electrostatic potential from 7.2 V (purple) to 28.8 V (red) on isosurface of the MEM charge density at 1.5 e/Å^3.

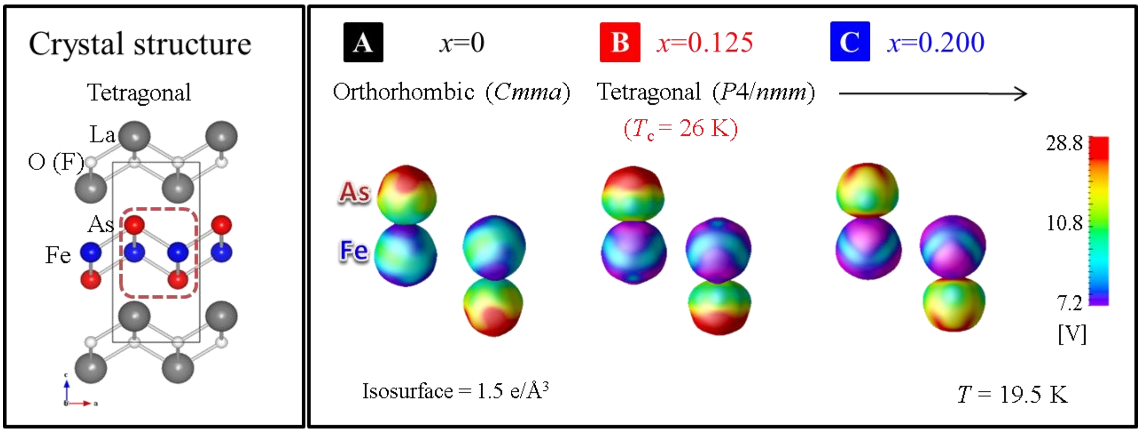

S6. Variation of the electrostatic potential distribution on the FeAs layer as a function of temperature at *x* = 0.125 and *x* = 0.200

In order to discover structural characteristics of the superconducting phase at the electronic interplay level, we have visualized the electrostatic potential as a function of temperature. In **Fig. S6**, the electrostatic potential of the As ion at x = 0.125 showed a radical change below the 62 K, compared to that of the x = 0.200. To investigate the change of electrostatic potential distribution around the As layer caused by electronic polarization of the As ion, we have visualized the space distribution of the electrostatic potential between the LaO and the FeAs layer. **Fig. S7** shows the equipotential map on the (040) plane, including the As atom and the Fe atom. At x = 0.125, asymmetric distribution around the As layer drastically increased below 42 K. On the contrary, the electrostatic potential distribution of x = 0.200 didn't change as temperature decreased, and it showed almost symmetric distribution. Variation of the electrostatic potential distirbution on the As ion is in good agreement with the result of the Fe-As bond mean-square relative displacements as a function of temperature reported by Zhang *et al*. (Zhang *et al*., 2010).

Figure S6 Tridimensional electrostatic potential distribution of the FeAs layer as a function of temperature at x = 0.125 and x = 0.200. The electrostatic potential was colored by the value from 7.2 V (purple) to 28.8 V (red) on isosurface of the MEM charge density at 1.5 e/Å^3.

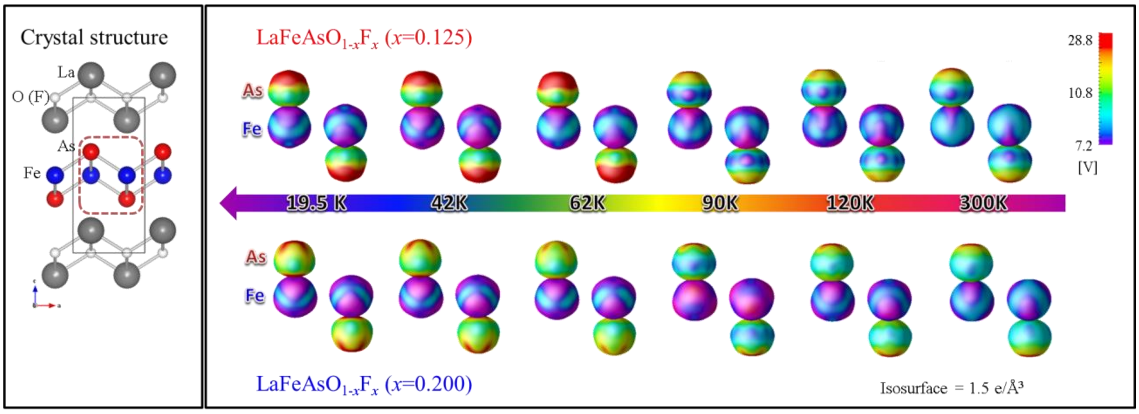

Figure S7 Two-dimensional mapping of electrostatic potential as a function of temperature for x = 0.125 and x = 0.200. The equipotential map shows the (040) plane including the As atom and Fe atom. It was displayed from -20.88 V (red) to -17.28 V (violet). The equipotential lines were drawn from -25.20 V to 0 V with an interval of 0.72 V.

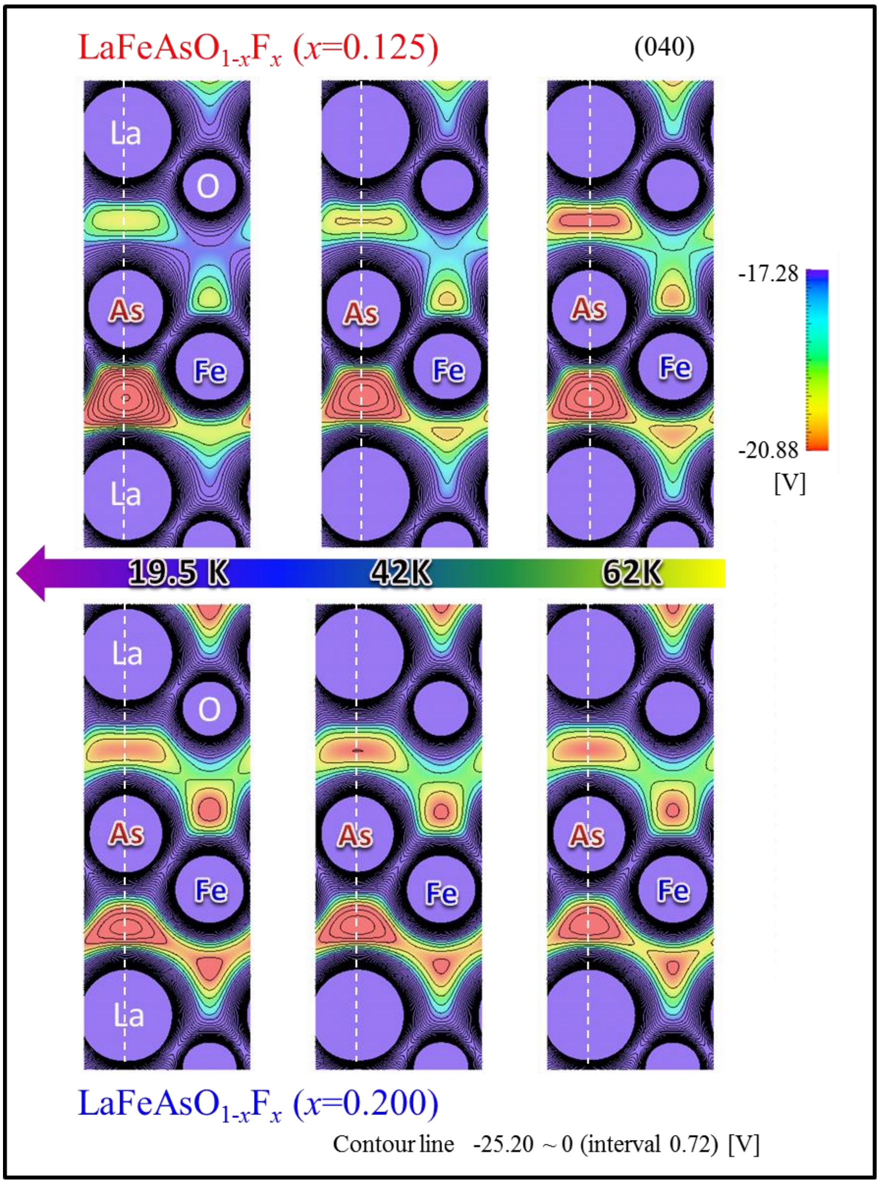

S7. Degradation of electronic polarization of the As ion at *x* = 0.200

In order to investigate the reason why the electronic polarization feature of the As ion decreased at *x* = 0.200, we have further visualized the electrostatic potential between the As ion and the nearest-neighboring La ion at 19.5 K as shown in **Fig. S8**. The electrostatic potential between the As ion and the nearest-neighboring La ion shows low value in the non-superconducting phase, irrespective of the bond length; the minimum value of the electrostatic potential was -13.5 V at *x* = 0.125 in the superconducting phase, whereas that was -15.3 V and -16.3 V at *x* = 0, and -16.1 V at *x* = 0.200 where the superconductivity disappeared. Depressed value of the electrostatic potential between the As ion and the nearest-neighboring La ion can be ascribed to the enhancement of covalency between both ions. This idea is supported by the fact that the electron density of the La-As bond is enhanced in non-superconductors; The electron density at the minimum potential point between the As ion and nearest-neighboring La ion was 0.053(2) e/Å^3 for *x* = 0.125, 0.119(3) e/Å^3 and 0.162(3) e/Å^3 for *x* = 0, and 0.104(3) e/Å^3 for *x* = 0.200. We suppose that enhanced covalent characteristics between the nearest-neighboring La and the As ions can disturb the separation of the electrostatic potential distribution on the As ion caused by confined electrons in the Fe layer.

Figure S8 The electrostatic potential of the plane including the As ion and the nearest-neighboring La ion at 19.5 K. The color of the two-dimensional map was disposed from -20.88 V (red) to -14.40 V (violet). The equipotential lines were drawn from -23.04 V to 2.88 V with an interval of 1.44 V. The lattice planes show the (040) and (200) for *x* = 0 with the orthorhombic structure, and the (1-10) for *x* = 0.125 and *x* = 0.200 with the tetragonal structure.

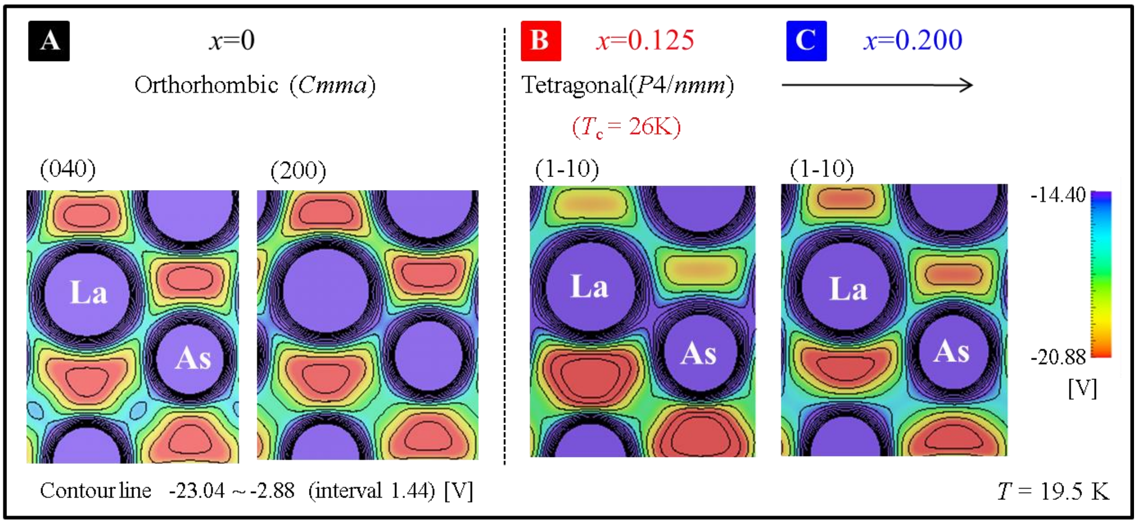

Supplement: Supplementary file 1 [file m-01-00155-sup1.pdf]
